# Elucidating how the saprophytic fungus *Aspergillus nidulans* uses the plant polyester suberin as carbon source

**DOI:** 10.1186/1471-2164-15-613

**Published:** 2014-07-21

**Authors:** Isabel Martins, Diego O Hartmann, Paula C Alves, Celso Martins, Helga Garcia, Céline C Leclercq, Rui Ferreira, Ji He, Jenny Renaut, Jörg D Becker, Cristina Silva Pereira

**Affiliations:** Instituto de Tecnologia Química e Biológica, Universidade Nova de Lisboa, Av. da República, 2780-157 Oeiras, Portugal; Instituto de Biologia Experimental e Tecnológica (iBET), Av. da República, 2781-901 Oeiras, Portugal; Proteomics Platform, Centre de Recherche Public - Gabriel Lippmann, Belvaux, Luxembourg; Cancer Genomics Research Laboratory, Division of Cancer Epidemiology and Genetics, National Cancer Institute, NIH, DHHS, (previously, the Scientific Computing department, Samuel Roberts Noble Foundation, USA, 8717 Grovemont Circle, 20877 Gaithersburg, MD USA; Instituto Gulbenkian de Ciência, Rua da Quinta Grande 6, 2780-156 Oeiras, Portugal

**Keywords:** *Aspergillus nidulans*, β-oxidation, Cutinase, Long chain fatty acids, Suberin, Whole-genome profiling

## Abstract

**Background:**

Lipid polymers in plant cell walls, such as cutin and suberin, build recalcitrant hydrophobic protective barriers. Their degradation is of foremost importance for both plant pathogenic and saprophytic fungi. Regardless of numerous reports on fungal degradation of emulsified fatty acids or cutin, and on fungi–plant interactions, the pathways involved in the degradation and utilisation of suberin remain largely overlooked. As a structural component of the plant cell wall, suberin isolation, in general, uses harsh depolymerisation methods that destroy its macromolecular structure. We recently overcame this limitation isolating suberin macromolecules in a near-native state.

**Results:**

Suberin macromolecules were used here to analyse the pathways involved in suberin degradation and utilisation by *Aspergillus nidulans*. Whole-genome profiling data revealed the complex degrading enzymatic machinery used by this saprophytic fungus. Initial suberin modification involved ester hydrolysis and ω-hydroxy fatty acid oxidation that released long chain fatty acids. These fatty acids were processed through peroxisomal β-oxidation, leading to up-regulation of genes encoding the major enzymes of these pathways (*e.g. faaB* and *aoxA*). The obtained transcriptome data was further complemented by secretome, microscopic and spectroscopic analyses.

**Conclusions:**

Data support that during fungal growth on suberin, cutinase 1 and some lipases (*e.g.* AN8046) acted as the major suberin degrading enzymes (regulated by FarA and possibly by some unknown regulatory elements). Suberin also induced the onset of sexual development and the boost of secondary metabolism.

**Electronic supplementary material:**

The online version of this article (doi:10.1186/1471-2164-15-613) contains supplementary material, which is available to authorized users.

## Background

Plant lipid polymers, particularly cutin and suberin, are the third most abundant of the plant polymers [1], yet the least understood since the underlying polyester structure remains partially unresolved. High recalcitrance is an inherent property of their molecular structure and hallmark monomers are often identified in soils [2, 3]. Filamentous fungi are key recyclers and compose nearly 75% of the soil microbial biomass [4] but their role in the turnover (biodegradation) of plant polyesters remains largely overlooked [5]. Suberin is a structural component of the secondary cell walls in specialised tissues, namely in the phellem of tree barks and subterranean organs [5, 6] and in the endodermis of roots [7]. Cutin, together with waxes (*viz*. cuticle), covers the cell walls in the epidermis of aerial tissues [8, 9]. Biosynthesis of either suberin or cutin is developmentally regulated and triggered as response to infection or wounding, among other challenges [8–13]. These lipid hydrophobic barriers constrain apoplastic water and solute translocation, physically strengthen the cell wall and, might also play roles in plant–pathogen interactions [14, 15].

**Figure 1:**
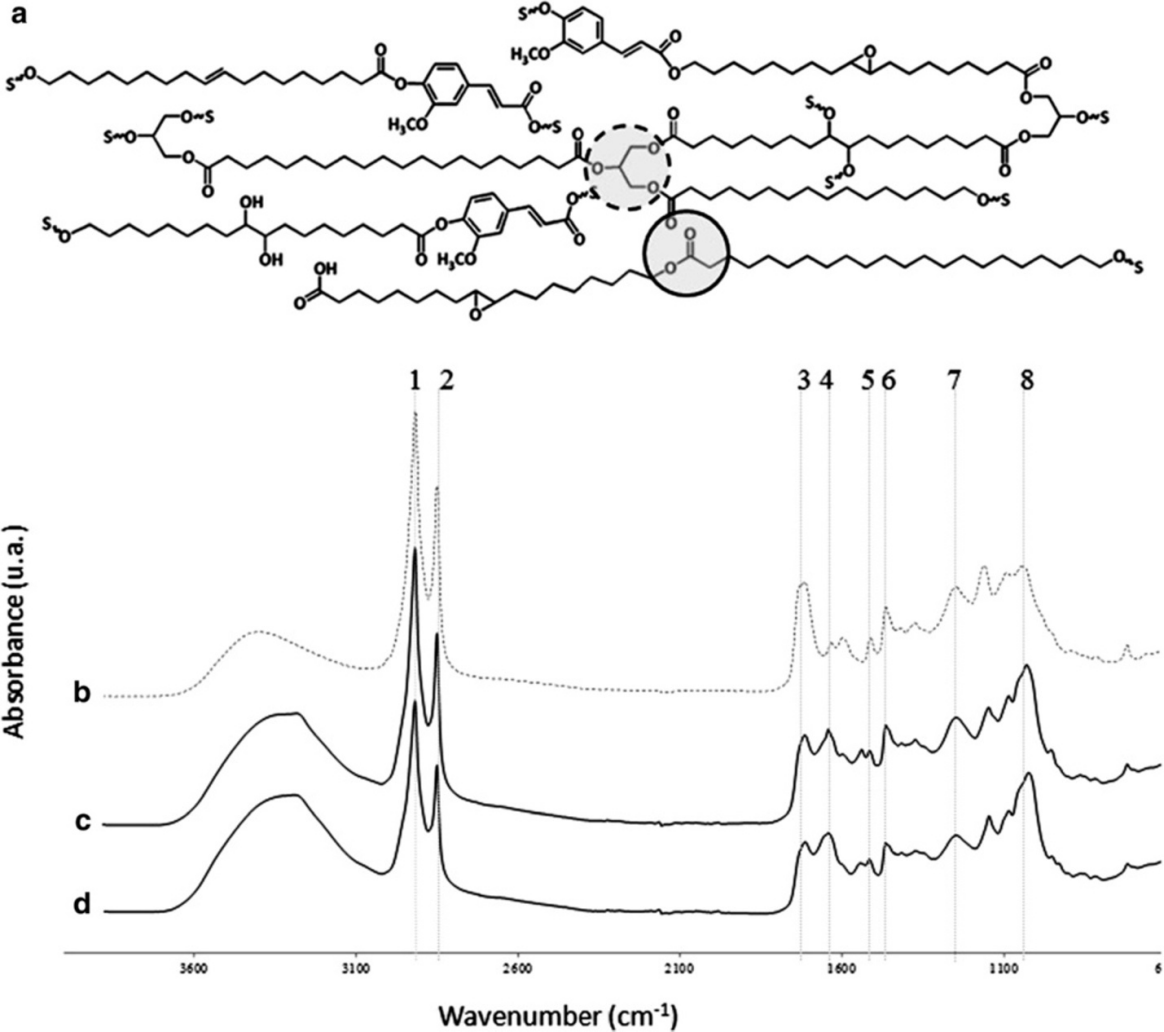
**Schematic view of suberin (a), showing the linear aliphatic ester bonds and the acylglycerol ester bonds (continuous and dashed circles, respectively) and the ATR-FTIR spectra of isolated suberin (a), namely untreated (b) and recovered at the first (c) and the last (d) time points of incubation with** ***Aspergillus nidulans*** **.** Major peaks, which can be almost exclusively assigned to suberin, are at 2921 cm^-1^ (1), 2851 cm^-1^ (2) and 1737 cm^-1^ (3). The remaining peaks are simultaneously assigned to the fungal cell wall and either to suberin [1158 cm^-1^ (7) and 1635 cm^-1^ (4)], lignin [1511 cm^-1^ (5) and 1424 cm^-1^ (6)] or to polysaccharides and lignin [1034 cm^-1^ (8)].

In general, both suberin and cutin contain fatty acids (FAs), ω-hydroxy FAs and glycerol but suberin also contains high levels of α,ω-dicarboxylic acids, ferulic acid and fatty alcohols and its saturated aliphatics have longer chain lengths than in cutin (>C20 and C16-18, respectively) (Figure 1a) [5, 10]. These composing monomers are linked essentially through acylglycerol or linear aliphatic ester bonds, building a three-dimensional network [16, 17]. Fungal degradation of plant polyesters has been reported to involve the activity of carboxylesterases, namely cutinases [18–20]. Despite high complexity and redundancy, it is well established that FAs can be used by filamentous fungi via β-oxidation leading to the production of acetyl-CoA [21, 22]. Downstream pathways, such as the glyoxylate and the tricarboxylic acid cycles and gluconeogenesis, enable the fungus to use FAs as sole carbon and energy sources.

In plants, suberin is ingrained between a primary and a tertiary cell wall and cross-linked to the other cell wall polymers, hence its extraction usually destroys its chemical skeleton [5]. This is still a major obstacle [5] and has restrained most biodegradation studies to the use of cutin films [23–25] and/or mixtures of nearly non-esterified suberin or cutin samples [26–28]. However, near-native suberin can be efficiently extracted from cork (outer bark of *Quercus suber* L.) through selective hydrolysis of acylglycerol ester bonds but leaving most linear aliphatic ester bonds intact [29–32] (Figure 1). In the present contribution we demonstrate that suberin macromolecules could be used as sole carbon source by *Aspergillus nidulans*. During fungal growth, synergetic action of cutinases, lipases and long chain fatty alcohol modifying enzymes released suberin long chain fatty acids (LCFAs) that were metabolised via peroxisomal β-oxidation. Other major alterations included the onset of sexual development and the boost of secondary metabolism.

## Methods

### Chemicals

If not otherwise stated chemicals were of the highest analytical grade and purchased from Sigma Aldrich (USA). Suberin was extracted from cork using cholinium hexanoate as previous described [30], afterwards freeze dried and kept at -20°C until used. The same process was used to recover the residual suberin upon fungal incubation. All suberin samples were analysed using attenuated total reflectance Fourier transform infrared spectroscopy (ATR-FTIR) as previously described [30, 32]. The selected ATR-FTIR spectra should be regarded as representative (ten replicates).

### Culture conditions

Fungal cultures with a density of *A. nidulans* (FGSC A4) conidia of 10^5^*per* mL (5 mL six-well plates, minimal media [33] containing 1% w/v glucose) were grown for two days (27°C, dark, 90 rpm) for the establishment of a mycelia mat against the bottom of the wells (control). Afterwards, old media were replaced by minimal media containing 0.1% w/v of suberin as sole carbon source, and cultures were grown, in the same conditions, for additional two, four, six or fifteen days. At each time point mycelia and the corresponding media filtrate were recovered and preserved at -80°C [33]. Additional control cultures were prepared containing 0.1% w/v of C16 glyceryl tripalmitate or C8 octyl octanoate (contain acylglycerol ester and linear ester bonds, respectively) instead of suberin. Controls were processed as described above.

### Microscopic analyses

Scanning Electron Microscopy (SEM) analysis of lyophilised fungal mycelia were performed using an analytical field emission gun scanning electron microscope (FEG-SEM: JEOL 7001 F with Oxford light elements EDS detector) operated at 5-10 kV. After the LIVE/DEAD assay [34] (evaluates culture viability) and the safranin staining (detects the extracellular matrix typical of fungal biofilm [35]), mycelia were evaluated using a DM5500 B microscope (Leica) under fluorescent (49 DAPI and N21 filter sets) or white light, respectively. 10x and 63x magnification objectives were used, respectively and images were captured with a DFC420 C camera (Leica). In all tests the selected micrographs should be regarded as representative.

### Secretome analyses

Extracellular protein was recovered from the culture filtrates using denaturing precipitation conditions [33]. 25 μg of protein (bovine serum albumin equivalents accordingly to the Bradford protocol) were loaded in a precast gel (Criterion™ XT precast 1D gel 4-12% Bis-Tris) and allow to migrate for 1 cm. The gel was stained with Instant Blue (Gentaur BVBA), sliced into 5 bands; (each cut into 1-2 mm cubes), first reduced, alkylated and de-stained, then digested by trypsin (Promega). The peptides were first desalted and finally fractionated by reverse phase separation in an Ultimate 3000 NanoLC System coupled to a LTQ-OrbiTrap Elite MS that was operated in data-dependent mode, automatically switching between MS and MS2, using XCalibur software. Data was processed in Mascot using Proteome Discoverer by searching against the SwissProt Fungi (released on January 21^st^, 2013) and NCBI databases. Protein identification was done using a set of pre-defined filters and a minimum confidence of 95%. Full details in Additional file 1. Glycerol in the culture media filtrates (40 times concentrated, duplicates) was analysed by chromatography as reported before [36]. Quantification detection limits were 0.01 g · L^-1^. The additional control cultures (see above) were also analysed.

### RNA isolation and cRNA preparation

Total RNA was isolated from fungal mycelia (grounded to powder using mortar and pestle in liquid nitrogen) using the RNeasy Plant Mini Kit (QIAGEN) and further purified by ethanol precipitation [37]. Quantification and purity of RNA were determined on a NanoDrop 1000 Spectrophotometer (Thermo Scientific) and RNA integrity analysed using an Agilent 2100 Bioanalyser with a RNA 6000 Nano Assay (Agilent Technologies). Fragmented and biotinylated cRNA was obtained by following GeneChip 3’ IVT Express Kit protocols. Briefly, 100 ng of total RNA were used for the synthesis of cDNA, which was further *in vitro* transcribed into labelled cRNA. After purification and fragmentation, the size distribution of the cRNA and fragmented cRNA were assessed in an Agilent 2100 Bioanalyzer with a RNA 6000 Nano Assay.

### Microarray processing

The custom array FungiANC (Affymetrix) contains a total of 20,012 transcripts from the genetic information of *A. nidulans* and *Neurospora crassa* available at the Broad Institute database (http://www.broadinstitute.org) and is based on a Perfect Match-only (PM) design with 11 micron feature size. Each transcript is represented by 11 probes (25-mer each). See full details in Additional file 2. The array processing was performed accordingly to Affymetrix GeneChip protocols (biological triplicates). A total of 200 μl of the hybridisation mixture containing 10 μg of fragmented cRNA was hybridised on arrays for 16 hours at 45°C. Standard post hybridisation washes and double-stain protocols (FS450_0001) were used on an Affymetrix GeneChip Fluidics Station 450, in conjunction with the GeneChip Hybridisation Wash and Stain Kit (Affymetrix). Arrays were scanned on an Affymetrix GeneChip Scanner 3000 7G. Array quality parameters were analysed by Expression Console Software (Affymetrix) for Robust Multiarray Averaging summarised data and confirmed to be in the recommended range. The data discussed in this publication have been deposited in NCBI’s Gene Expression Omnibus [38] and are accessible through GEO Series accession number GSE54427 (http://www.ncbi.nlm.nih.gov/geo/query/acc.cgi?acc=GSE54427).

### Microarray data analysis

DNA-Chip Analyzer (dChip) 2010 (http://www.dchip.org) was used applying a probeset mask file considering only the *A. nidulans* probes (9675 transcripts). The normalised CEL intensities of the 12 arrays (Invariant Set Normalisation Method [39, 40]), were used to obtain model-based gene expression indices based on a Perfect Match-only model [39, 40]. dChip Log2 expression data were imported into R v2.13.0. Differentially expressed genes (analysis was carried out with Bioconductor LIMMA package, http://www.bioconductor.org), included only the probe sets with adjusted *p*-value ≤0.01 and fold-change (FC) ≥2. See full details in Additional file 1.

### Quantitative real-time PCR analysis (*q*RT-PCR)

Based on the sequences of *A. nidulans* genes (*Aspergillus* Genome Database, http://www.aspergillusgenome.org/), all oligonucleotide pairs were designed using the GeneFisher2 web tool (http://bibiserv.techfak.uni-bielefeld.de/genefisher2) and produced by Thermo Fisher Scientific (see Additional file 1 for the list of the oligonucleotides used in this study). The *q*RT-PCR analysis was performed in a CFX96 Thermal Cycler (Bio-Rad), using the SsoFast EvaGreen Supermix (Bio-Rad), 250 nM of each oligonucleotide and the cDNA template equivalent to 1 ng of total RNA, at a final volume of 10 μl per well, in three technical and three biological replicates. The PCR conditions were: enzyme activation at 95°C for 30 s; 40 cycles of denaturation at 95°C for 10 s and annealing/extension at 59°C for 15 s; and melting curve obtained from 65°C to 95°C, consisting of 0.5°C increments for 5 s. Data analyses were performed using the CFX Manager software (Bio-Rad). The expression of each gene was taken as the relative expression in pair-wise comparisons of consecutive time points. The expression of all target genes was normalised by the expression of the 60S ribosomal protein L33-A gene, AN2980, selected as internal control due to its constant levels in all time points.

### Functional annotation

Annotations for all the genes represented on the FungiANC genome array were obtained from the Broad Institute database (http://www.broadinstitute.org) and the *Aspergillus* Genome Database (AspGD, http://www.aspgd.org). See full details in Additional file 2. The differentially expressed genes for each biological condition were classified using the FungiFun web annotation tool [41] and the Functional Catalogue (FunCat). The significant hits (*p*-value ≤0.05) were defined using the identities present on the chip as the background.

## Results & discussion

### *Aspergillus nidulans*transcriptome on suberin - enrichment analysis

**Figure 2:**
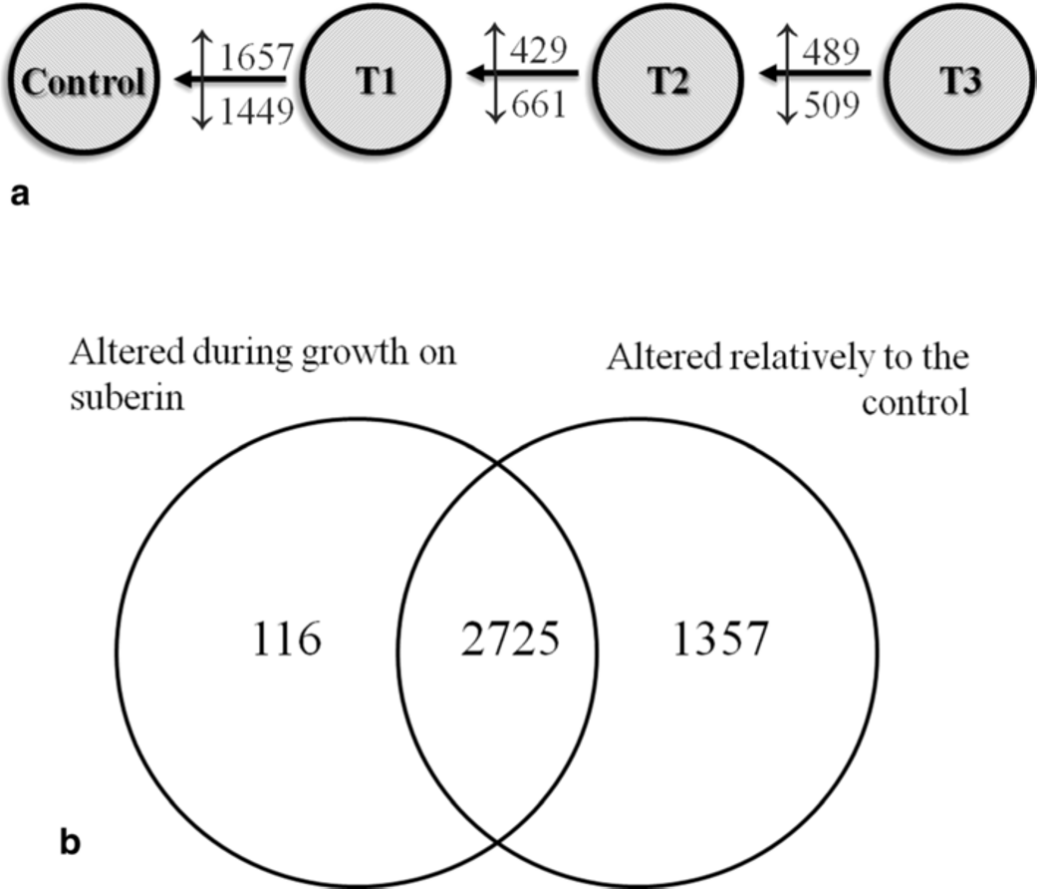
**Number of differentially expressed genes during** ***Aspergillus nidulans*** **growth on suberin in pair-wise comparisons of consecutive times (a), discriminating the up- (↑) and the down- (↓) regulated genes, and Venn diagram highlighting the number of genes that were responsive only at the first time point on suberin when compared with the control (b).**

Pair-wise comparisons were used to identify differentially expressed genes (adjusted *p*-value < 0.01 and |FC| > 2) between the control (grown on glucose) and during growth on suberin for two, four or six days (hereafter defined as first, mid and last time points, respectively) Additional file 2. Within the differentially expressed genes (4198 constituting nearly half of the transcripts in the microarray), 32% (1357 genes) can be specifically associated with the switch of the substrate (control *vs* first time point) and were enriched in functional categories associated with the metabolism of alkanes, alkenes, alkanals and alkanols (MIPS 01.20.05.03) and the oxidation of fatty acids (MIPS 2.25), as well as cellular sensing and response to external stimulus (MIPS 33.11) and cell type differentiation (MIPS 43), among others (Figure 2b, Additional file 3). Pair-wise comparison of consecutive time points, henceforward systematically used, showed that among the enriched functional categories at the mid time point some were associated with increased nutrient starvation response (MIPS 32.01.11) and alterations in fatty acid metabolism (MIPS 01.06.05), along with major alterations in cell cycle (MIPS 10) and cell fate (MIPS 40) Additional file 3. In addition, those enriched at the last time point revealed *e.g.* an increased stress response (MIPS 32.01) and development of ascospores (MIPS 43.01.03.09). In particular, the degradation/modification of exogenous ester compounds (MIPS 32.10.09) can be associated with cleavage of ester bonds in suberin. The intensity of the major peak assigned to ester bonds (1737 cm^-1^ which can be exclusively assigned to the C = O stretch of ester groups) in the ATR-FTIR spectra of suberin decreased significantly after fungal incubation (Figure 1b).

### Autolysis and primary metabolism

Autolysis occurred after switching from glucose to suberin media. Major up-regulation of *pepJ* (AN7962, Table 1) and up-regulation of *prtA* (AN5558) resulted in accumulation of the encoded proteins in the secretome (Table 2). Both proteases play a role in the degradation of empty hyphae during starvation [42, 43]. Out of the 25 protein species identified in the secretome, ten are involved in cell wall remodelling events typical of autolysis and show, in general, high consistency with the transcriptome data (Table 2). Several other cell wall remodelling genes related with autolysis were up-regulated along cultivation: AN9042, *mutA* (AN7349), AN7613, AN2690 and AN8392, *chiB* (AN4871) [44, 45], *engA* (AN0472) [46] and *nagA* (AN1502) [44] (Table 1 and Additional file 2). Glucuronan lyase A gene (AN0012) has been reported to be up-regulated in *A. nidulans* during starvation [47] but here its expression level at the first time point was exceedingly higher (Table 1). The encoded protein specifically breaks β-1,4-glucuronans, which are rarely found in the cell walls of *Aspergilli* [48] and absent in those of cork [6]. Hence, the functional role of AN0012 remains largely unclear. Few genes related with apoptosis (AN7500 and AN5712) [49, 50] or autophagy (AN2876 and AN5174) [51] were responsive along the incubation, suggesting that they played only a minor role during the fungus growth on suberin Additional file 2. As typically reported in *A. nidulans* after imposition of severe carbon starvation conditions, several putative major facilitator superfamily (MFS) transporter genes (AN9232, AN8084, AN6778, AN8502) [47, 52] underwent major up-regulation (Table 1). The last two genes belong to uncharacterised secondary metabolite clusters [53], thus probably coding for specific transmembrane transporters. Some other MFS genes (AN5860, *mstE* and AN4180) and one auxin efflux carrier superfamily gene (AN8018) were amongst those showing major down-regulation (Table 1). *mstE* is induced in the presence of several repressing carbon sources and is dependent on the function of CreA (AN6195) [54]. Up-regulation of *creA*, the carbon catabolite repressor A [55], at the first time point (Table 3) might be explained by a self-repression mechanism [56, 57]. Gluconeogenesis activation might have occurred as suggested by the up-regulation of the pathway transcription activator (*acuM*, AN6293) and of phosphoenolpyruvate carboxykinase gene (*acuF*, AN1918) which, together with the fructose 1,6-bisphosphatase gene (*acuG*, AN5604, not differentially expressed in the suberin media), regulate this pathway [58] (Table 4, Additional file 2). The 4-aminobutyrate (GABA) shunt is inactive during gluconeogenesis [59], likely coordinated with major down-regulation of putative glutamate decarboxylase gene (AN7278) at the first time point (Table 1). Major up-regulation of the fermentation transcription activator *alcR* (AN8978, Table 1) at the last time point occurred but the aldehyde dehydrogenase gene (AN0554) [60] was not differentially expressed Additional file 2.

**Table 1:** **List of the differentially expressed genes showing the highest fold changes (FCs) in pair-wise comparison of consecutive time points during** ***Aspergillus nidulans*** **growth on suberin**

First time point of suberin incubation (between the control and second day of incubation on suberin)			Mid time point of suberin incubation (between the second and fourth days of incubation on suberin)			Last time point of suberin incubation (between the fourth and sixth days of incubation on suberin)		
FC	Gene #	Description	FC	Gene #	Description	FC	Gene #	Description
*Up-regulated*								
358.7	AN6000	Asperthecin polyketide synthase (*aptA*)	31.8	AN7369	GMC oxidoreductase	23.5	AN9230	Monooxygenase associated with secondary metabolism
305.8	AN5309	Putative cutinase 1 (*cut1*)	22.0	AN10030	Putative alkaline serine protease	9.6	AN6778	MFS transporter
278.8	AN7962	Metalloproteinase (*pepJ*)	22.7	AN9224	Monooxygenase	8.6	AN10026	Oxidoreductase
252.5	AN9042	Mutanase	21.7	AN9493	Putative acetyltransferase (*ngn12*)	7.0	AN11202	Putative DMATS-type aromatic prenyltransferase
107.4	AN0012	Glucuronan lyase A	20.1	AN9227	Dioxygenase associated with secondary metabolism	6.6	AN8392	Melibiase subfamily
91.6	AN7812	Putative sterigmatocystin biosynthesis protein (*stcN*)	19.4	AN5348	Fatty acyl-CoA reductase	6.6	AN8106	Dioxygenase associated with secondary metabolism
90.1	AN7349	Mutanase (*mutA*)	18.7	AN7613	Putative chitinase	6.4	AN8473	RNA polimerase II transcription factor
58.5	AN2623	Acyl-CoA:6-aminopenicillanic-acid-acyltransferase (*aatA*)				5.8	AN8978	Regulatory protein (*alcR*)
46.7	AN11013	Probable sterigmatocystin biosynthesis P450 monooxygenase (*stcL*)	16.4	AN2690	Putative β-1,3-glucanase	5.5	AN8084	MFS transporter
35.0	AN3931	Meiotic expression up-regulated protein 14 (*pilB*)				5.0	AN8520	protein required for terrequinone A biosynthesis (*tdiE*)
33.4	AN6835	NADPH-cytochrome P450 reductase	16.2	AN9232	MFS transporter	5.0	AN3369	Zn_2_-Cys_6_ transcription factor (*clrB*)
32.8	AN7811	Putative sterigmatocystin biosynthesis protein (*stcO*)	16.6	AN9234	Oxidoreductase associated with secondary metabolism	4.9	AN6747	C2H2 type zinc finger transcription factor
33 < FC < 87		Predicted proteins and poorly characterised genes	17 < FC < 147		Predicted proteins and poorly characterised genes	4 < FC < 8		Predicted proteins and poorly characterised genes
AN1532, AN8995, AN6476, AN9301, AN7235, AN2330, AN4825, AN2913, AN0461, AN8656, AN1666, AN0488, AN1952			AN6020, AN4970, AN7958, AN7957, AN5292, AN5319, AN8037, AN1155, AN2400, AN2710, AN7655, AN8162, AN9235, AN11163, AN1946			AN7419, AN7092, AN2779, AN3881, AN7091, AN7395, AN8955, AN2658, AN2859, AN1719, AN2376, AN5422		
*Down-regulated*								
-39.5	AN0399	Nitrate transporter (*nrtB*)	-18.6	AN7824	Probable sterigmatocystin biosynthesis P450 monooxygenase (*stcB*)	-13.1	AN2583	Glyceraldehyde 3-phosphate dehydrogenase
-35.9	AN1008	Nitrate transporter (*crnA*)	-18.1	AN8356	Alcohol dehydrogenase	-9.8	AN1666	Nucleolar GTP-binding protein 2
-35.4	AN1006	Nitrate reductase (*niaD*)	-14.1	AN7804	Putative sterigmatocystin biosynthesis monooxygenase (*stcW*)	-8.6	AN2861	Putative F-box protein
-26.1	AN7539	Hydrophobin	-12.6	AN7818	Probable sterigmatocystin biosynthesis P450 monooxygenase (*stcF*)	-6.3	AN5228	NADH:flavin oxidoreductase/NADH oxidase
-24.4	AN7392	Choline transporter Hnm1	-11.8	AN7807	Putative sterigmatocystin biosynthesis protein (*stcT*)			
			-11.5	AN7815	Fatty acid synthase subunit α	-5.6	AN4180	MFS transporter
			-11.4	AN5860	MFS monosaccharide transporter (*mstE*)			
-24.1	AN2859	Dihydrodipicolinate synthetase	-11.2	AN7825	Putative sterigmatocystin biosynthesis polyketide synthase (*stcA*)	-5.6	AN10619	Glutamate decarboxylase
			-11.2	AN4135	Stearoyl-CoA desaturase (*sdeB*)			
-23.9	AN4131	Na^+^/H^+^ antiporter	-11.1	AN7814	Fatty acid synthase subunit β	-5.5	AN7169	Flavohemoprotein (*fhbA*)
-23.8	AN7278	Glutamate decarboxylase	-10.8	AN7806	Versicolorin reductase			
-21.7	AN4119	MFS multidrug transporter	-10.6	AN3763	Siderochrome-iron transporter	-5.5	AN3264	MFS multidrug transporter
-19.0	AN3776	MFS transporter	-10.0	AN7811	Putative sterigmatocystin biosynthesis protein (*stcO*)			
-61 < FC < -18		Predicted proteins and poorly characterised genes	-28 < FC < -10		Predicted protein and poorly characterised genes	-16 < FC < -5		Predicted protein and poorly characterised genes
AN2595, AN7214, AN5305, AN8081, AN6946, AN4108, AN7378, AN9220, AN6128, AN8981, AN3333, AN6930, AN4128, AN6932, AN8670			AN7397, AN3251, AN8544, AN6661, AN7817, AN8314, AN2722, AN0169, AN7812, AN7809, AN7915, AN11013			AN2571, AN9310, AN7949, AN5489, AN0421, AN0461, AN9102, AN9378, AN7960, AN3305, AN0728, AN0247, AN0288, AN8974, AN0838, AN8544, AN3314

**Table 2:** **List of the extracellular protein species identified in the secretome of** ***Aspergillus nidulans*** **at the last time point of growth on suberin**

Gene number	Description	MW [kDa]	calc. pI	SignalP ^a^	SwissProt		NCBI		Microarray data*		
					Total peptides	Cov (%)	Total peptides	Cov (%)	First ^c^	Mid ^c^	Last ^c^
*Plant Polymer Degrading Enzymes*											
AN5309	Cutinase (Cut1)	22.4	7.3	Y	3	12.21	2	9.09	**305.8**	**-3**	1.5
AN8046	Triacylglycerol lipase	31.3	4.6	Y	-^b^	-^b^	7	47.28	**10.4**	-1.6	**2.7**
AN3613	β-1,4-xylanase (XlnA)	24.1	6.3	Y	4	36	4	36	**6.5**	-2.2	-1.1
AN7401	β-1,4-endoxylanase (XlnE)	37.7	5.5	Y	-^b^	-^b^	3	11.83	-1.3	-1.4	1
AN1818	β-1,4-xylanase C (XlnC)	35.4	5.2	Y	5	20.49	-^b^	-^b^	1.2	-1.2	-1.3
AN8477	β -1,4-xylosidase	60.3	5.4	N	-^b^	-^b^	4	9.71	1.6	1.1	1.1
AN2828	β-glucosidase L (BglL)	77.8	4.8	Y	14	21.71	12	22.52	-1.2	-1.3	**2.4**
*Fungal Development*											
AN0472	β-1,3-endoglucanase A (EngA)	97.8	5.7	Y	-^b^	-^b^	6	8.49	**5.8**	1.1	1.2
AN7950	β-1,3-endoglucosidase (EglC)	46.7	4.6	Y	10	28.39	10	28.39	-^d^	-^d^	-^d^
AN4825	β-1,3-glucosidase	96.5	5.5	Y	-^b^	-^b^	11	17	**42.5**	1.1	1.2
AN2395	β-glucuronidase	68.5	4.8	Y	-^b^	-^b^	14	28.06	**7.4**	-1.4	1.2
AN4871	Chitinase B (ChiB)	44.2	5.6	Y	-^b^	-^b^	34	80.65	**12.7**	1.4	1.1
AN2017	α-glucosidase (AgdA)	109.6	5.2	Y	-^b^	-^b^	6	8.17	-1.5	-1.9	**2.3**
AN8445	Aminopeptidase Y	53.7	6.8	Y	-^b^	-^b^	6	19.68	1.8	-1.5	1.6
AN2366	Serine protease	25.4	4.4	Y	-^b^	-^b^	3	17.27	-^d^	-^d^	-^d^
AN5558	Alkaline serine protease (PrtA)	42.2	5.3	Y	8	32.75	7	32.75	**5.4**	1.3	1.1
AN7962	Metalloproteinase (PepJ)	37.4	5.1	N	6	18.36	6	18.36	**278.8**	1.8	1.2
AN4245	Ceramidase	80	5	Y	-^b^	-^b^	7	14.8	1.6	1.5	1.8
AN9339	Catalase B (CatB)	79.1	5.1	Y	23	35.6	20	34.54	**-2.4**	1.8	1
*Miscellaneous*											
AN3351	Uncharacterised	63.2	5.1	Y	-^b^	-^b^	5	12.91	1.6	-1.4	-1.1
AN3246	Uncharacterised	22.3	6.4	N	-^b^	-^b^	4	29.8	2	-1.3	2
AN6273	Allergenic Asp F13	16.3	4.8	Y	-^b^	-^b^	3	32.91	-1	-1.5	-1.4
AN5879	Phosphatidylglycerol/phosphatidylinositol transfer protein	18.3	5	Y	9	53.25	9	53.25	1	1	-1
AN8979	Alcohol dehydrogenase I (AlcA)	36.9	6	N	5	25.57	5	25.57	**-15.1**	**-8.8**	1
AN8043	Uncharacterised	16.9	4.8	N	-^b^	-^b^	3	26.42	**-4**	-2	-1

^a^SignalP was used to predict secretion signals [67, 68] and ^b^not found in the database search. Corresponding microarray data are shown for comparison. ^*^Values highlighted in bold have |FC| > 2 and p-value < 0.01 in the microarray data; ^c^Fold changes (FCs) in pair-wise comparison of consecutive time points; ^d^not represented in the chip.

**Table 3:** ***q*** **RT-PCR analysis of a selected set of genes coding putative lipid hydrolysing enzymes or major regulatory proteins**

Carbon source	Gene	Encoded protein	***q***RT-PCR			Microarray*		
			First ^d^	Mid ^d^	Last ^d^	First ^d^	Mid ^d^	Last ^d^
Suberin	AN6195	CreA	8.3	-2.3	-1.9	**4.3**	-1.4	-1.6
	AN1052	VeA	2.5	-1.0	-1.0	**2.6**	-1.3	1.0
	AN7050	FarA	2.3	-1.7	1.3	**2.8**	-1.7	1.3
	AN5309	Cut1	119.9	-3.1	1.8	**305.8**	**-3.0**	1.5
	AN7541	Cut2	-1.5	-1.0	1.5	-1.3	-1.1	1.1
	AN7180	Cutinase	1.2	1.1	1.9	1.3	-1.2	-1.1
	AN5267	FaeC	3.2	-2.1	1.7	**3.0**	**-2.4**	1.1
	AN2697	Putative lipase^a^	-1.8	1.4	1.8	1.6	-1.7	-1.1
	AN5777	Putative lipase	2.9	1.0	1.7	**2.9**	-1.2	1.6
	AN8046	Putative lipase	4.1	-1.1	2.3	**10.4**	-1.6	**2.7**
	AN8900	Putative lipase^b^	-1.8	1.7	1.5	1.6	1.6	-1.1
	AN4748	Uncharacterised protein^c^	31.8	-1.8	-3.8	**24.6**	-1.7	**-4.5**
Glyceryl tripalmitate	AN5309	Cut1	1.4	36.4	-7.8			
	AN7541	Cut2	2.7	-3.8	-1.3			
	AN7050	FarA	3.7	-1.7	-1.7			
	AN8046	Putative lipase	8.2	2.3	-19.4			
Octyl octanoate	AN5309	Cut1	3.3	14.0	-1.6			
	AN7541	Cut2	4.1	-1.2	-3.0			
	AN7050	FarA	2.7	-1.3	-1.4			
	AN8046	Putative lipase	2.8	5.8	-11.7			

Values represent the relative expression of selected genes in pair-wise comparisons of consecutive time points. The expression of each gene was normalised by the expression of the 60S ribosomal protein L33-A gene (AN2980).

^*^Values highlighted in bold have |FC| > 2 and *p*-value < 0.01 in the microarray data; ^a^contains feruloyl esterase and tannase domains, high homology with feruloyl esterase B in *N. crassa*; ^b^orthologue of *A. niger* An09g02270, which encodes a triacylglycerol lipase; ^c^orthologue of *S. cerevisiae* NOP6, which is necessary for rRNA-binding protein required for 40S ribosomal subunit biogenesis.^d^Fold changes (FCs) in pair-wise comparison of consecutive time points. Corresponding microarray data are shown for comparison.

**Table 4:** **List of** ***Aspergillus nidulans*** **differentially expressed genes (pair-wise comparison of consecutive time points), putatively involved in suberin degradation**

Gene number	Description (gene name)	Microarray data ^*^			Sub-cellular location ^a^	Number of predicted binding sites for ***farA***
		First ^d^	Mid ^d^	Last ^d^		
*Lipid Hydrolysis & Transport*						
*Cutinases*						
AN5309	Cutinase 1 (*cut 1*)	**305.8**	**-3.0**	1.5	Extracellular	4^b^, 4^c^
AN7180	Cutinase	1.3	-1.2	-1.1	Extracellular	2^b^, 2^c^
AN7541	Cutinase (*cut2*)	-1.3	-1.1	1.1	Extracellular	3^b^, 3^c^
AN10346	Cutinase	1.5	-1.4	-1.3	Extracellular	2^b^
*Other extracellular esterases*						
AN1799	Triacylglycerol lipase	**22.3**	-1.9	1.6	Extracellular	1^b^
AN2602	Lipase/esterase	**16.9**	-2.0	**-3.3**	Extracellular	0^b^
AN8046	Putative triacylglycerol lipase	**10.4**	-1.6	**2.7**	Extracellular	0^b^
AN6773	Putative triacylglycerol lipase	**5.1**	**13.1**	-1.4	Extracellular	1^b^
AN4573	Hydrolase (ester bonds)	**3.5**	2.2	3.2	Extracellular	0^b^
AN5777	Triacylglycerol lipase	**2.9**	1.2	1.6	Extracellular	0^b^
AN6464	Hydrolase (ester bonds)	2.3	-1.5	1.0	Extracellular	1^b^
AN7158	Hydrolase (ester bonds)	**-2.2**	-1.1	-1.1	Extracellular	0^b^
AN5321	Triacylglycerol lipase	-1.5	**2.8**	1.1	Extracellular	2^b^
AN1433	Triacylglycerol lipase	**-6.1**	-1.9	1.8	Extracellular	1^b^
AN3037	Carboxylesterase	**-7.2**	1.3	**2.4**	Extracellular	1^b^
AN1792	Hydrolase (ester bonds)	**-15.9**	-1.4	**2.3**	Extracellular	1^b^
*Transporters*						
AN6581	ABC drug exporter (*atrF*)	**12.0**	-1.3	**-4.1**	Membrane	1^b^
AN8813	ABC transporter	**8.6**	**-2.2**	1.2	Membrane	2^b^
AN2300	ABC multidrug transporter (*atrD*)	**5.1**	-1.1	-1.7	Membrane	0^b^
AN6369	ABC transporter	**4.8**	-1.6	-1.2	Membrane	0^b^
AN0771	ABC transporter	**4.7**	-1.3	-1.9	Membrane	0^b^
AN8892	ABC multidrug transporter	**3.6**	**-3.5**	-1.3	Membrane	1^b^
AN8489	ABC multidrug transporter	**2.1**	-1.3	1.2	Membrane	0^b^
*Other genes*						
AN0623	Long chain fatty alcohol oxidase	1.7	**13.7**	-1.2	Unknown	2^b^
AN6795	Putative hydrophobic surface binding protein A	**9.2**	-2.5	1.1	Extracellular	1^b^
*Fatty Acids β-Oxidation*						
*Transport to the peroxisome/mitochondria*						
AN6279	Carnitine acetyltransferase (*acuJ*)	**3.0**	-1.6	-1.3	Peroxisome/mitochondria	2^b^, 2^c^
AN5356	Carnitine/acyl-carnitine carrier (*acuH*)	**2.0**	**-2.6**	-1.0	Mitochondria	1^b^, 1^c^
AN0257	Peroxisomal ATP carrier protein (*antA*)	**4.2**	-1.4	-1.2	Peroxisome	3^b^, 3^c^
*β-oxidation cycle*						
AN5646	Acetyl-CoA acyltransferase	**4.6**	-1.7	-1.5	Peroxisome	1^b^, 1^c^
AN5698	Acetyl-CoA acyltransferase	-1.2	**-2.2**	1.3	Mitochondria	0^b^
AN1699	Acyl-CoA dehydrogenase	**8.9**	-1.1	-1.6	Peroxisome	3^b^, 3^c^
AN7320	Acyl-CoA dehydrogenase	**2.4**	**-2.1**	1.4	Peroxisome	1^b^
AN9162	Acyl-CoA dehydrogenase	**-2.0**	-1.1	1.4	Mitochondria	0^b^
AN6761	Acyl-CoA dehydrogenase	**-2.8**	1.4	1.2	Mitochondria	0^b^
AN2762	Acyl-CoA dehydrogenase	**-2.2**	-1.5	1.7	Mitochondria	1^b^
AN12335	Acyl-CoA dehydrogenase (*acdA*)	**-2.7**	1.6	1.2	Peroxisome	1^b^, 1^c^
AN0824	Acyl-CoA dehydrogenase (*scdA*)	**2.1**	-1.4	1.1	Mitochondria	3^b^, 3^c^
AN8280	Acyl-CoA synthetase (*faaB*)	**7.9**	**-2.6**	-1.4	Peroxisome	2^b^, 2^c^
AN5192	Acyl-CoA synthetase (*fatA*)	**3.0**	**-2.8**	1.1	Peroxisome	2^b^
AN4397	Acyl-CoA synthetase (*fatD*)	1.2	**-2.3**	-1.6	Peroxisome	1^b^
AN10512	β-ketoacyl-CoA thiolase (*mthA*)	1.9	**-2.2**	-1.8	Mitochondria	1^b^
AN6752	Long chain fatty acyl-CoA oxidase (*aoxA*)	**5.7**	-1.4	-1.4	Peroxisome	3^b^, 3^c^
AN2999	NADP-isocitrate dehydrogenase (*idpA*)	**3.5**	-1.5	-1.4	Peroxisome/mitochondria	1^b^
AN4688	Isovaleryl-CoA dehydrogenase (*ivdA*)	1.1	-1.5	**3.0**	Mitochondria	0^b^
*Gluconeogenesis*						
AN6293	Transcription activator (*acuM*)	**2.1**	-1.6	1.6	Cytosol	2^b^
AN1918	Phosphoenolpyruvate carboxykinase (*acuF*)	**6.8**	**-3.8**	-1.2	Cytosol	1^b^
AN5604	Fructose 1,6-bisphosphatase (*acuG*)	1.9	-1.8	1.0	Cytosol	2^b^
*Glyoxylate cycle*						
AN5634	Isocitrate lyase (*acuD*)	2.9	-2.2	-1.7	Peroxisome	1^b^,1^c^
AN6653	Malate synthase (*acuE*)	-1.2	1.2	-1.2	Peroxisome	2^b^,2^c^
*Regulatory genes*						
AN7050	Zn_2_-Cys_6_ transcription factor (*farA*)	**2.8**	-1.7	1.3	Nucleus	0^b^
AN1425	Zn_2_-Cys_6_ transcription factor (*farB*)	**4.2**	-1.8	1.0	Nucleus	1^b^
AN1303	Zn_2_-Cys_6_ transcription factor (*scfA*)	**-2.0**	**-2.8**	-1.1	Nucleus	0^b^
AN0689	Zn_2_-Cys_6_ transcription factor (*facB*)	**5.4**	**-2.4**	-1.4	Nucleus	0^b^

^*^values highlighted in bold have |FC| > 2 and *p*-value < 0.01 in the microarray data; ^a)^ Sub-cellular location was attained using Pedant Database (http://pedant.gsf.de). ^b^the number of predicted binding sites for *farA* was manually searched according to the conserved sequence 5’-CCTCGG or its reverse complement sequence (5’-CCGAGG) within 1 Kb of the upstream region of the start codon of listed genes; ^c^number of predicted binding sites as previous reported [22]. ^d^Fold changes (FCs) in pair-wise comparison of consecutive time points.

The transcriptional regulation of the assimilatory nitrate system in *A. nidulans* differs in high availability or limiting glucose conditions [61]. AreA is the major nitrogen regulatory protein, however, under glucose limiting conditions, the nitrogen status-sensing regulator AreB controls the expression of the nitrate catabolic genes [61, 62]. Major down-regulation of nitrate reductase *niaD* (AN1006) and of nitrate transporters (AN1008, AN0399) (Table 1) was consistent with the down-regulation of nitrite reductase *niiA* (AN1007) and the up-regulation of *areB* (AN6221) Additional file 2. At the last time point, the inducible nitric oxide-detoxifying flavohaemoglobin gene (AN7169, *fhbA*) showed major down-regulation (Table 1). This gene is co-regulated with *niaD* and *niiA*, yet its expression is AreA-independent [63, 64].

### Development and secondary metabolism

**Figure 3:**
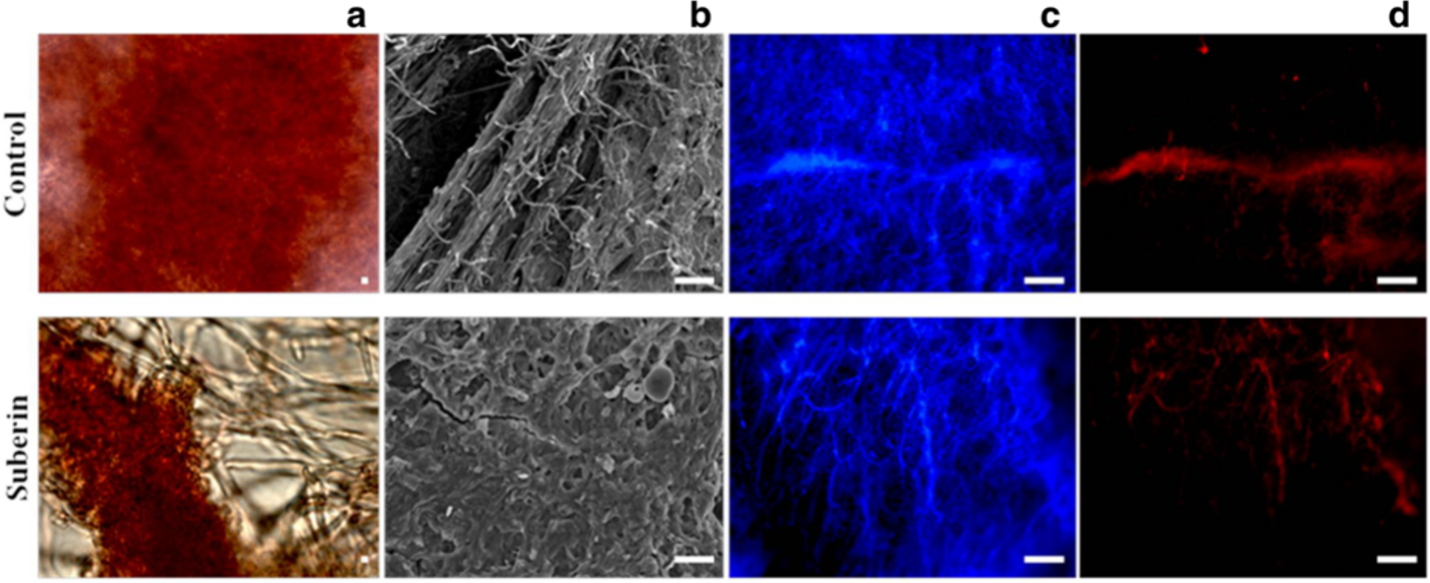
**Microscopic analysis of** ***Aspergillus nidulans*** **mycelia in controls (top panels) or on suberin (bottom panels) at the first time point, showing the red safranin stain (scale bar: 137 μm) (a), the hyphal morphology (detected by SEM, scale bar: 10 μm) (b), and the total (c) and the dead (d) hyphae, shown in blue (calcofluor white stain) and red (propidium iodide stain) (scale bar: 1000 μm).** Only the controls showed the typical features of fungal biofilms, namely the extracellular matrix stained with safranin and the hyphal alignment. Total and dead hyphae were alike in controls and on suberin.

During growth on suberin the mycelia mat surrounded the water-insoluble substrate, disrupting the fungal biofilm formed in the control, *i.e.* loss of hyphae alignment and disruption of the extracellular polysaccharide matrix (Figure 3). The expression levels of some *A. nidulans* orthologs of *A. fumigatus* genes coding in important pathways of biofilm formation were consistent with the loss of biofilm morphology [65], including major down-regulation of hydrophobin gene (AN7539) (Table 1).

**Figure 4:**
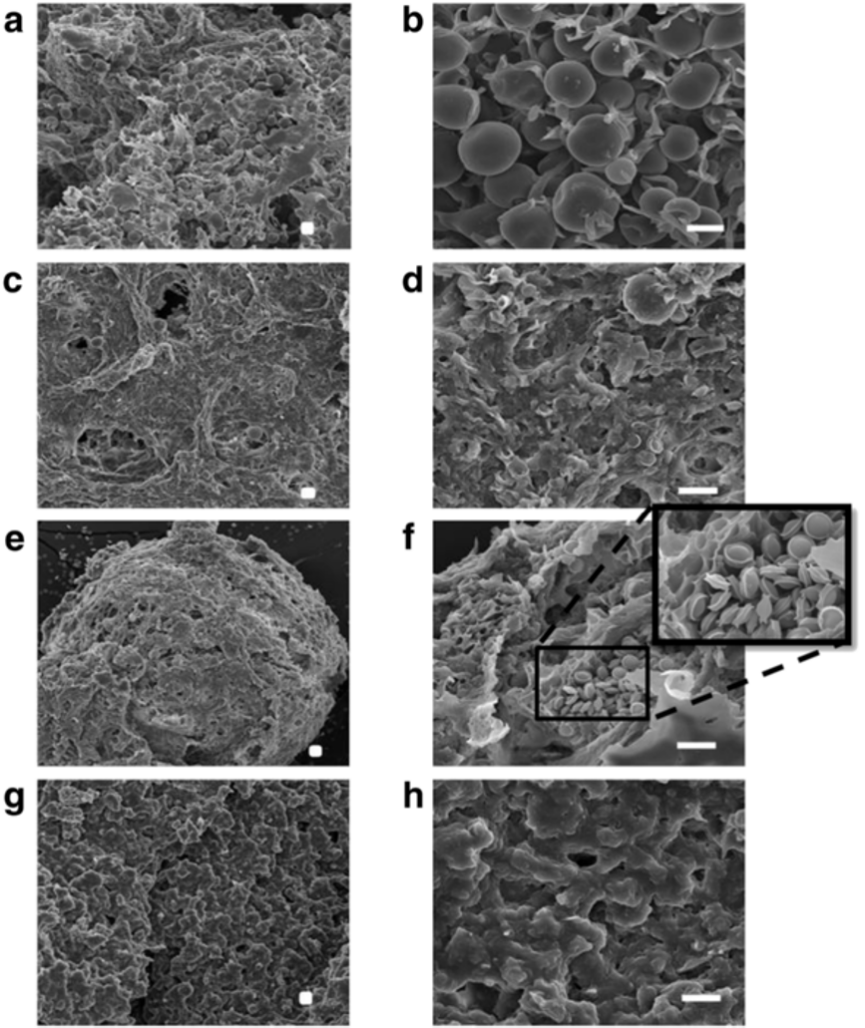
**SEM images of** ***Aspergillus nidulans*** **during growth on suberin.** Hülle cells were detected at the first time point **(a, b)**, few ascospores were detected at the last time point **(c, d)**, and numerous ascospores **(f)** and cleistothecia **(e)** were detected after fifteen days. Untreated suberin (control) is also shown **(g, h)**. Scale bar: 10 μm.

Sexual development in *A. nidulans* was induced during growth on suberin, similar to that observed before on FAs [22]: Hülle cells and few ascospores were detected at the first and last time points, respectively; whereas cleistothecia and numerous ascospores were observed only after fifteen days of cultivation (Figure 4). The breach of cleistothecia might explain the few ascospores detected at the last time point. In agreement, *mutA* (mutanase associated with the use of glucose reserves during the formation of Hülle cells [66]) and AN10030 (alkaline serine protease involved in the biosynthesis of the ascopore cell wall [67, 68]) underwent major up-regulation at first and mid time points, respectively. In addition, *MAT1* (AN2755) and *MAT2* (AN4734) genes, which encode the transcription factors considered the master switchers of sexual development in *A. nidulans* [69, 70], were up-regulated at the first time point Additional file 2. The expression profile of the vast majority of genes associated with sexual development in *A. nidulans*, including *steA* (AN2290), was consistent with the onset of sexual development [71]. The few exceptions included genes signalling response to nitrogen/carbon limitation [61], namely down- and up-regulation of sexual development activators (*csnB,* AN4783 and *noxA*, AN5457) and repressors (*silG,* AN0709; *cpcA,* AN3675 and *rosA,* AN5170), respectively Additional file 2.

The expression of genes regulating fungal growth and development is known to be coordinated with those coding in the biosynthesis of asperthecin, penicillin and sterigmatocystin [72]. Moreover, exogenous addition of mannans to the growth media (oligosaccharides that might be released during autolysis) increases penicillin production in *Penicillium* sp. [73]. In fact, genes in asperthecin (AN6000, *aptA*), penicillin (AN2623) and sterigmatocystin (AN7812, AN11013 and AN7811) clusters were amongst those more profoundly affected at the first time point (Table 1). Analyses of the expression levels of the other in-cluster genes confirmed these findings, *e.g.* up-regulation of genes in sterigmatocystin cluster included the *aflR* regulator (AN7820) [74], the polyketide synthase (AN7825) and the fatty acid synthase genes (AN7814 and AN7815) Additional file 2. At the mid time point, sterigmatocystin biosynthesis probably decreased, since genes encoding the synthase (AN7825) and some auxiliary enzymes (AN7804, AN7806, AN7811, AN7818 and AN7824) become major down-regulated (Table 1). Suberin also induced major alterations in numerous genes coding in uncharacterised clusters (AN9234, AN9227, AN9230, AN11202, AN8106 and AN8520). Overall, 65 secondary metabolite gene clusters, out of the 71 estimated by now in this fungus [53], were responsive but only in a few clusters the synthase gene was largely affected. Supplementation of the growth media with suberoylanilide hydroxamic acid induced several secondary metabolite synthases in *A. niger* [75] and potato suberin augmented the diversity of the secondary metabolites biosynthesised by *Streptomyces* sp. [76].

Twelve genes involved in the control of development and carbon metabolism, as well as in suberin degradation, were selected to validate the microarray data by *q*RT-PCR (Table 3). The tested genes included four genes which putatively encode lipid hydrolysing enzymes (AN7541, AN7180, AN2697 and AN8900) that showed FC ≤ 2 in the microarray. All the analysed genes, with the exception of AN2697, showed a profile of expression along the cultivation similar to that of the microarray data.

### Degradation of suberin: lipid hydrolysis

Several genes encoding polyester hydrolysing enzymes were induced during the fungus growth on suberin (Table 4). Data made apparent that Cut1 played the major role in suberin initial degradation: the encoding gene (AN5309) was extensively up-regulated (Table 1) and the enzyme was present in the secretome (Table 2). Cut1 hydrolyses aliphatic polyesters [77] and potato suberin [18, 19, 26]. FarA and FarB are major transcription activators of genes involved in FAs utilisation [22]. Deletion of *farA* (AN7050) eliminates induction of a number of genes by both small chain fatty acids (SCFAs) and LCFAs, while deletion of *farB* (AN1425) eliminates SCFAs induction [22]. As expected, *farA* and *farB* were both up-regulated at the first time point (Table 4). The down-regulation of *scfA* on suberin (Table 4, AN1303, which encodes a similar transcription factor and its deletion leads to *farB* deleted mutant phenotype [22]) might be explained by its repression under nitrogen limiting conditions [64]. With the exception of *cut1*, the expression levels of the other three cutinase genes were kept at basal levels (AN10346) or underwent minor alterations (*cut2* - AN7541 and AN7180) (Tables 3 and 4). FarA regulation of *A. nidulans* cutinase genes might be similar to that reported in *Fusarium solani* [26]. In the pathogenic fungus, *cut1* is strongly induced by cutin monomers under the regulation of CTF1α - which has 70% similarity with FarA and the same binding motif, CCTCGG - but *cut2* and *cut3* are expressed at basal levels. Similar regulation was noticed in media containing octyl octanoate or glyceryl tripalmitate as sole carbon sources (Table 3). In the suberin media major up-regulation of *cut1* occurred at an earlier time point probably because it contains *ca.* 4 wt% of soluble “cutin-like” monomers [30, 78]. Glycerol could not be detected in the suberin culture filtrates likely because 80-90% of the acylglycerol bonds in suberin were hydrolysed during its extraction (Figure 1a) [31]. In agreement, glycerol catabolic genes [79], namely glycerol kinase (AN5589, *glcA*) and FAD-dependent glycerol 3-phosphate dehydrogenase (AN1396) were not differentially expressed. When glyceryl tripalmitate was used as sole carbon source, glycerol was detected, namely 0.063 ± 0.003 and 0.0201 ± 0.0018 g⋅L^-1^ at the first and the last time point, respectively.

Only Cut1 and AN8046, putative lipid hydrolysing enzymes, could be detected in the secretome (Table 2). Gene expression data corroborate the hypothesis that *farA* regulates *cut1* and AN8046 during *A. nidulans* utilisation of suberin (Table 4), as well as octyl octanoate or glyceryl tripalmitate (Table 3) as sole carbon sources. The *farA* ortholog gene of *A. oryzae* regulates not only *cutL* (cutinase gene) but also the putative lipid hydrolysing enzyme genes *mdlB* (ortholog of AN8046) and *tglA* (70% homology with AN10346) [80]. It also regulates *hsbA* (ortholog of AN6795) that encodes hydrophobic surface binding protein probably involved in the recruitment of CutL to the FAs surface. AN6795 was also stimulated by suberin (Table 4). In addition, during *Fusarium oxysporum* growth on wheat oil, the transcription factor *ctf1* regulates both *cut1* and *lip1* [81], which is amongst the lipase genes more strongly induced [82] (*n.b.* Lip1 shows high homology to AN8046 protein).

Several other putative lipase genes, namely AN2602, AN6464, AN5777, AN4573, AN1799, AN6773, in addition to AN2697 and AN8900 (FC < 2, Table 3) were also stimulated by suberin (Table 4). Their regulation was variable, except AN4573 (increased along the incubation) and AN6773 (increased at first and mid time points). The latter, as well as AN1799 and AN5321, have been associated with unresolved secondary metabolite gene clusters in *A. nidulans* [53] and likely are not involved in suberin degradation.

Eleven genes encoding ABC transporters carrying transmembrane domains were up-regulated at the first time point. ABC transporters are generally assumed to be involved in multidrug resistance, yet more recent studies have shown their physiological significance *e.g.* in oxidative stress response, pathogenicity and excretion of siderophore peptide breakdown products [83]. A role in penicillin secretion was proposed for the *A. nidulans* AtrD transporter, which belongs to the subfamily ABC-C [84]. *atrD* (AN2300) up-regulation at the first time point agrees with major up-regulation of penicillin synthase gene (Table 1). Up-regulation of numerous ABC-G transporter genes (AN6581, AN8813, AN6369, AN0771, AN8892 and AN8489) might imply a possible involvement in the transmembrane transport of suberin hydrolysed monomers. The function of this subfamily of transporters remains largely unknown with putative roles in *e.g.* excretion of hydrophobic and/or lipid molecules [85]. In addition, suberin possibly stimulated the formation of eisosomes playing a role in the endocytosis of lipid cargos [86], as suggested by the major up-regulation of *pilB* (AN3931) (Table 1).

Hydrolysed suberin LC fatty alcohols (Figure 1a) need further modification before entering FAs β-oxidation pathways. ω-Hydroxy fatty acid oxidation was probably mediated by NADPH-cytochrome P450 reductase fusion enzyme (AN6835), as well as by LC fatty alcohol oxidase (AN0623). Both encoding genes were up-regulated during growth on suberin (Tables 1 and 4). The first enzyme might catalyse the oxidation of the LC fatty alcohols to carboxylic acids and of mid-chain saturation functionalities to vic-diols [87, 88]. LC fatty alcohol oxidases have been shown to catalyse the oxidation of ω-hydroxy fatty alcohols in *Candida cloacae* [89] and the encoding genes display usually a peroxisomal targeting sequence, notwithstanding their cellular localisation in *A. nidulans* remains unknown. Due to different substrate specificities and/or cellular compartmentalisation, possibly AN6835 and AN0623 enzymes have acted sequentially during *A. nidulans* growth on suberin. The hypothesis that LC fatty alcohols undergo modification by LC fatty alcohol oxidase in the peroxisome merits further analysis.

### Degradation of suberin: β-oxidation

Current understanding of FAs utilisation in *A. nidulans* indicates significant complexity and redundancy in β-oxidation pathways [21, 22, 90]. FAs are activated by FA-CoA synthetases to their acyl-CoA derivates, which are processed by FA-CoA oxidases or dehydrogenases. Each round of β-oxidation produces a chain-shortened fatty-acyl-CoA (which undergoes further rounds) and an acetyl-CoA, which is channelled into the tricarboxylic acid or glyoxylate cycles. In addition, β-oxidation substrates are actively transported across the mitochondria and/or the peroxisome membrane by carnitine shuttles, ADP/ATP carriers or ABC transporter proteins. Suberin stimulated two peroxisomal FA-CoA synthetase genes, namely *faaB* (AN8280) and *fatA* (AN5192) (Table 4) but none of the well characterised mitochondrial ones (*e.g. facA* (AN5626) and *pcsA* (AN5833), Additional file 2). It seems that hydrolysed suberin monomers were essentially processed *via* peroxisomal β-oxidation pathways (Table 4) and that, as previously suggested, FaaB is the major peroxisomal FA-CoA synthetase, while the remaining ones (FatA-D (AN5192, AN5877, AN6649, AN4397), FaaA (AN6114) and AN4659) display high functional redundancy [91]. Activation of peroxisomal β-oxidation agrees with the up-regulation of *antA,* a peroxisomal ATP carrier (AN0257) and of *acuJ*, a mitochondrial/peroxisomal carnitine acetyltransferase (AN6279) (Table 4). Uncharacterised transporters for the peroxisomal translocation of activated LCFAs have been suggested [22]; justifying the basal expression levels of *pxa1* and *pxa2* (AN10078 and AN1014, ABC transporter proteins).

Suberin stimulated the expression of *aoxA* (AN6752) but not *aoxB* (AN6765), both encoding peroxisomal fatty-acyl-CoA oxidases (Table 4) but AoxA plays the major role during growth on LCFAs [92]. Deletion of *aoxA* leads to growth impairment on LCFAs but not on SCFAs or very long chain fatty acids (VLCFAs) [21]. *aoxA* stimulation is consistent with the observation that suberin hydrolysis releases three times more LCFAs than VLCFAs [30, 78]. Only three peroxisomal fatty-acyl-CoA dehydrogenase genes (out of seven) were responsive at the first time point, namely *acdA* (AN12335), AN1699 and AN7320 genes (Table 4). Deletion of either *acdA* or AN7320 has not led to any growth impairment on FAs, suggesting that the encoded enzymes display high redundancy [92]. *Botrytis cinerea* BC1G_13535 gene, which displays 78% of homology with AN1699, was amongst the highest up-regulated genes coding in FAs β-oxidation during *Lactuca sativa* infection [93]. The hypothesis that this fatty-acyl-CoA dehydrogenase plays a major role in *A. nidulans* degradation of plant FAs calls for its functional characterisation.

LCFAs β-oxidation is shuttled between the peroxisomes and the mitochondria, typically when the produced chain-shortened fatty-acyl-CoA is a butyryl-CoA (C4) [92]. At the first time point, most mitochondrial fatty-acyl-CoA dehydrogenase genes were down-regulated (AN2762, AN6761 and AN9162), except *scdA* (AN0824) [22] that was up-regulated. The remaining mitochondrial β-oxidation genes, in general, decreased at the mid and/or last time points, including the well characterised *mthA*, a β-ketoacyl-CoA thiolase gene (AN10512), as well as *acuH,* a mitochondrial carnitine/acyl-carnitine carrier (AN5356) (Table 4). The only exception was *ivdA,* an isovaleryl-CoA dehydrogenase gene (AN4688) that was up-regulated, together with *mccB* (AN4687), at the last time point (Table 4). This might imply that the fungus started using leucine as a catabolic source; both genes are clustered with *mccA* (AN4690) in the leucine catabolic pathway [94].

The glyoxylate bypass is absolutely required for growth on carbon sources that produce acetyl-CoA and is dependent on isocitrate lyase (AcuD) and malate synthase (AcuE) activities. Transcription of *acuD* (AN5634) and *acuE* (AN6653) is regulated by FA and acetate induction *via* the FacB activator, but *facB* (AN0689) mutations do not prevent growth on FAs [22]. Up-regulation of *farA* and *farB* (FA-induced) and of *facB* (acetate-induced) led to up-regulation of *acuD* but not of *acuE* (Table 4). Previous studies have demonstrated that the imposition of several stresses might lead to both fluctuating mRNA and irregular protein expression levels in *A. nidulans* [95].

### Degradation of phenolic suberin

Release of ferulic acid during fungal growth on potato suberin has been suggested to involve the activity of feruloyl esterases (Fae) [5], notwithstanding a direct proof is still lacking. Ferulic acid release probably justifies the up-regulation of *faeC* (AN5267) (Table 3). Ferulic acid degradation might involve the activity of 2,3-dihydroxybenzoate carboxylyase [96], of which the encoding gene *dhbD* (AN6723) was up-regulated at the first time point (Additional file 2). Several downstream products have been reported in different fungal strains, however the associated enzymes remain largely unknown [96]. Nevertheless, final degradation of the formed aromatics might involve the homogentisate, benzoate or the salicylate pathways [97], and several genes coding in these pathways were found to be up-regulated (*e.g.* AN10950, *hmgA* (AN1897), *hpdA* (AN1899), *maiA* (AN1895), Additional file 2) in the suberin media.

### Degradation of other cell wall constituents

Several polysaccharides degrading enzymes were identified in the *A. nidulans* secretome on suberin at the last time point (Table 2, Additional file 4), in general, agreeing with the transcriptome data (Additional file 2). They included the β-glucosidase BglL (AN2828) and the xylanase XlnA (AN3613), of which the encoding genes were found up-regulated at the last and the first time points, respectively. The other β-glucosidase genes were up-regulated at the first (*bglM*, AN7396) and mid time points (*eglB* (AN5214) and AN3046) and could not be detected in the secretome. *xlnR* (AN7610), which encodes the xylanolytic/cellulolytic transcriptional activator, was up-regulated at the last time point (consistent with *creA* decrease [98]), notwithstanding the up-regulation of the *xlnA* and *xlnB* (AN9365) at the earlier time points. The additional polysaccharide degrading enzymes detected in the secretome were not consistent with the transcriptome data (Table 2), including XlnC (AN1818) and XlnE (AN7401). Probably the up-regulation of their corresponding genes occurred at an intermediate time point. The poor correlation between transcriptomic and proteomic data has been previously reported [95, 99], often related to mechanisms associated with mRNA turnover and/or efficiency of translation [100] or to transcription on demand of certain mRNA pools [101], among other reasons, including experimental noise [102]. Protein species grouped in the miscellaneous category (six, of which half have a predicted intracellular localisation) might be associated with cell lysis, as highlighted by the presence of alcohol dehydrogenase I (*alcA*, AN8979) (Table 2).

## Conclusions

**Figure 5:**
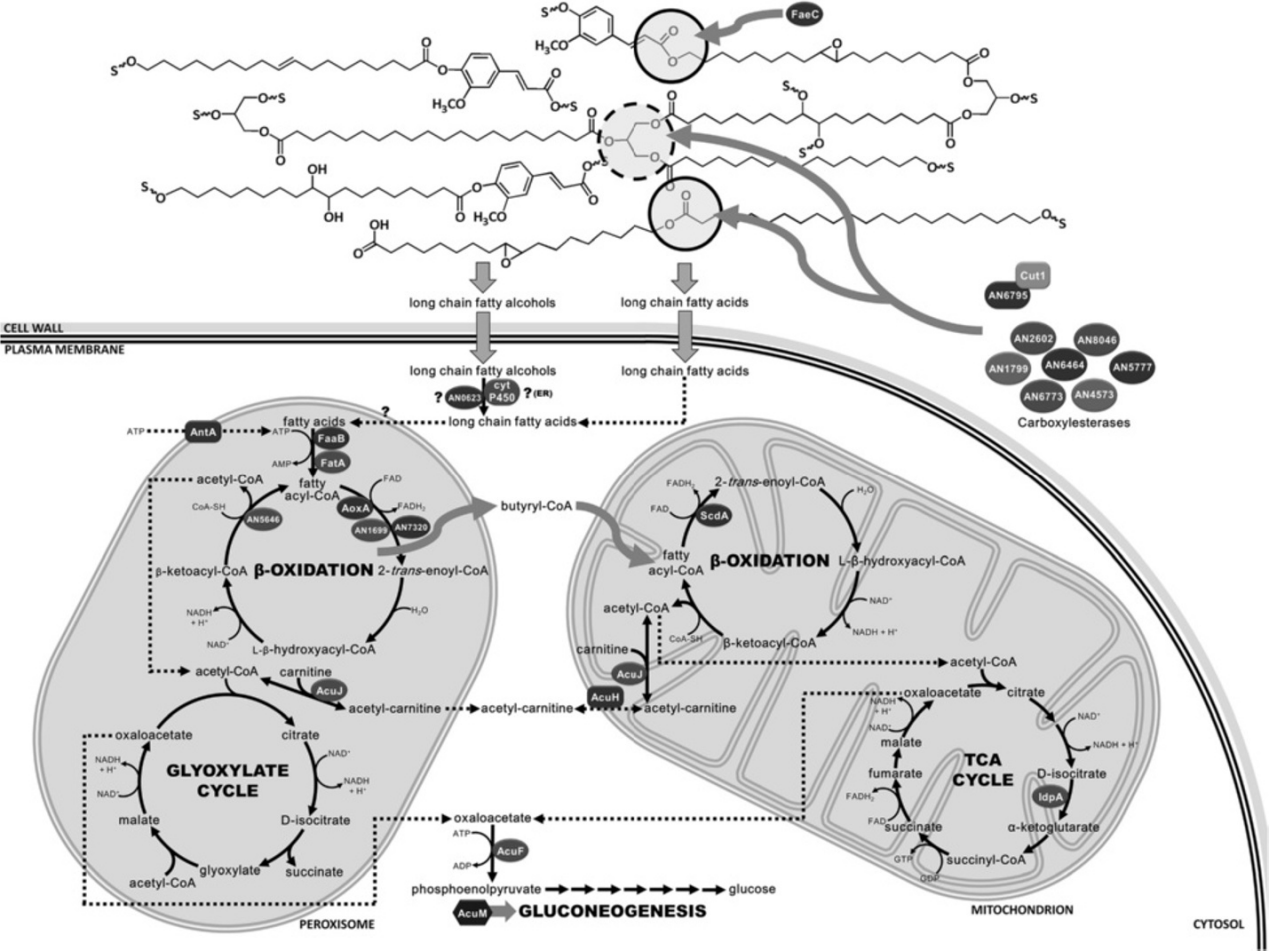
**Schematic view of putative suberin degradation and utilisation pathways in** ***Aspergillus nidulans*** **.** For sake of clarity, some steps and intermediates are omitted and only the proteins of which the encoding genes were up-regulated in the first and the mid time points are represented. Uncertainties in the cellular compartmentalisation or activity of the enzyme are indicated by question marks.

Previous studies on *A. nidulans* colonisation of cork revealed that suberin remained unaltered [103] probably because the outermost lignin-enriched cell wall layers hampered its degradation. Here we have shown that *A. nidulans* was able to utilise suberin macromolecules as sole carbon source (Figure 1b) and that the fungus also underwent sexual development (Figure 4) and boosted secondary metabolism (Table 1). We propose the suberin degradation and utilisation pathway in *A. nidulans*, as depicted schematically in Figure 5. Amongst the up-regulated genes encoding lipid hydrolysing enzymes only two were detected in the secretome, namely Cut1 and AN8046 (Tables 2, 3 and 4). In particular, out of the four cutinase genes, only *cut1* expression pattern was correlated to that of *farA*, similar to that described in plant pathogenic fungi [22, 81]. ω-Hydroxy fatty acid oxidation reactions (mediated by either NADPH-cytochrome P450 reductase or LC fatty alcohol oxidase, Tables 1 and 4), are likely involved in the modification of suberin LC fatty alcohols, even if their cellular compartmentalisation remains uncertain. The hydrolysed suberin monomers were essentially composed of LCFAs, hence activated to their corresponding acyl-CoA derivatives probably by FaaB; the major peroxisomal fatty acyl-CoA synthetase in this fungus [92]. Despite high functional redundancy of additional peroxisomal FA-CoA synthetase genes, *fatA* showed the highest up-regulation on suberin. Up-regulation of *aoxA* also occurred, agreeing with the idea that the encoded fatty-acyl-CoA oxidase plays a major role during growth on LCFAs [21]. In addition, among the peroxisomal fatty-acyl-CoA dehydrogenase genes up-regulated here, AN1699 underwent the highest up-regulation (Table 4), similar to that reported for its *B. cinerea* ortholog during plant infection [93]. The core binding sequence for FarA, typically overrepresented in the promoter region of genes required for growth on FAs, is not present in all related genes up-regulated during *A. nidulans* growth on suberin (Table 3). Some unknown regulatory elements are certainly yet to be discovered, further emphasised by the down-regulation of some lipid hydrolysing genes carrying the FarA recognition site.

## Availability of supporting data

The data sets supporting the results of this article are included within the article (and its additional files).

## Authors’ information

This research constitutes an important milestone of C. Silva Pereira’s team (established in 2008) that is addressing a major scientific question: *How fungi perceive and interact with the environment?* What makes our research approach distinctive is the merging of fundamental biology research with chemical expertise. Such multidisciplinary environment has inspired the development of a new extraction method for suberin - a structural component of the plant cell wall. Elucidating how fungi utilise and degrade plant polyesters strongly impacts on our understanding of plant-fungi interactions and nutrients cycling. Complementary to this research, we have reported that the plant polyester can be reconstituted as a material that combines bactericidal and antibiofouling properties. We are particularly interested in further elucidating plant polyesters physiological roles and to promote the clinical use of plant polyester based materials.

## Electronic supplementary material

Additional file 1: **Materials and Methods details.** (DOCX 26 KB)

Additional file 2: **List of the differentially expressed genes.** (XLSX 4 MB)

Additional file 3: **Functional categorisation of the genes significantly altered during suberin incubation.** (XLSX 34 KB)

Additional file 4: **Protein identification data.** (XLSX 26 KB)

## Abbreviations

FC: Fold change
FAs: Fatty acids
SCFAs: Small chain fatty acids
LCFAs: Long chain fatty acids
VLCFAs: Very long chain fatty acids
ATR-FTIR: Attenuated total reflectance Fourier transform infrared spectroscopy
SEM: Scanning electron microscopy
*q*RT-PCR: Quantitative real-time PCR analysis
MIPS: Munich Information Center for Protein Sequences.
