# Supplementary material for: Elucidating how the saprophytic fungus Aspergillus nidulans uses the plant polyester suberin as carbon source
Source: BMC Genomics. 2014 Jul 21;15:613. doi: 10.1186/1471-2164-15-613 (PMC4117967; doi:10.1186/1471-2164-15-613)
Supplement: Supplementary file 1 — Additional file 1: Materials and Methods details. (DOCX 26 KB) [file 12864_2014_6294_MOESM1_ESM.docx]

**Electronic Supplementary Information 4**

**Elucidating how the saprophytic fungus *Aspergillus nidulans* uses the plant polyester suberin as carbon source**

Isabel Martins,^a^ Diego O. Hartmann,^a^ Paula C. Alves,^a^ Celso Martins,^a^ Helga Garcia,^a^ Céline C. Leclercq,^b^ Rui Ferreira,^a^ Ji He,^c^ Jenny Renaut,^b^ Jörg D. Becker ^d^ and Cristina Silva Pereira ^a*^

^a^ Instituto de Tecnologia Química e Biológica, Universidade Nova de Lisboa, Av. da República, 2780-157 Oeiras, Portugal

^b^ Proteomics Platform, Centre de Recherche Public - Gabriel Lippmann, Belvaux, Luxembourg

^c^ Cancer Genomics Research Laboratory, Division of Cancer Epidemiology and Genetics, National Cancer Institute, NIH, DHHS, 8717 Grovemont Circle, Gaithersburg, MD, 20877, USA (previously, the Scientific Computing department, Samuel Roberts Noble Foundation, USA)

^d^ Instituto Gulbenkian de Ciência, Rua da Quinta Grande 6, 2780-156, Oeiras, Portugal

Corresponding author *

Cristina Silva Pereira

Instituto de Tecnologia Química e Biológica, Universidade Nova de Lisboa, Av. da República, EAN, 2781-901, Oeiras Portugal

Tel.+351 211157786

Fax. +351 214411277

E-mail: [spereira@itqb.unl.pt](mailto:spereira@itqb.unl.pt)

**Contents. Material and Methods details**.

**1)** Table S1. List of the designed *q*RT-PCR oligonucleotides (forward and reverse) used in the validation of the microarray data. Due to its constant expression in all conditions, the 60S ribosomal protein L33-A gene (AN2980) was selected as internal control....................................................................3

**2)** Details on MS-based secretome analyses:.....................................................................................................4

**Table S1**. List of the designed *q*RT-PCR oligonucleotides (forward and reverse) used in the validation of the microarray data. Due to its constant expression in all conditions, the 60S ribosomal protein L33-A gene (AN2980) was selected as internal control.

| **Gene Code (Name)** | **Forward Sequence (5′ to 3′)** | **Reverse Sequence (5′ to 3′)** |
| --- | --- | --- |
| AN1052 (*veA*) | TCAGAGCTCCCATCGACCA | GCCGGTCATCATGACCGAA |
| AN2697 | GATTACGAGGGTGAGATCCAGA | GGGATCTTGAAGGCAGGGAA |
| AN4748 | GAGTCAGGAGGAACAAGAGCA | GTAAGGGAGATTCCCGACGAA |
| AN5267 (*faeC*) | AGCCCACGGATATCGGTCA | AGCGAGCTTCCAACACCAA |
| AN5309 (*cut1*) | CGTCTCGATCAACGCCAGA | GAGTTTCAGGACATTGCAGGTA |
| AN5777 | CTTTCGGGGAACCAAGGGTA | CTGTAAGCGGGATTAGGGGTA |
| AN6195 (*creA*) | CATTCGCACGAGGATGAGGA | GCCAATGGCGTGTGGTCA |
| AN7050 (*farA*) | CCCTGAGATTCTGTCCAGCA | CGTCTTGGCTTGCATTATCCA |
| AN7180 | TCTACCCTTGGCTCCGACA | CATTTAGCGGCGAGGTCGA |
| AN7541 (*cut2*) | CAAACACAGGTGAAGCTTCCA | ACCAGCTAGAGAGACCGCTA |
| AN8046 | TCACAGACCAGGGGGCAA | TATCTGTCGTCGTCGGGGTA |
| AN8900 | TGGCGACAACATGTACTACGA | TTCCATACCGTCCTTTGATGGA |
| AN2980 | TCTACCGGGCTAAGCGTGA | CACGGACAGTAGCACCGAA |
| AN6542 (*actA*) | CTGGAAAGCGGTGGTATCCA | TGCATACGGTCGGAGATACC |

**Details on MS-based secretome analyses**

*Peptide desalting* and enrichment were achieved using a C18 pre-column (C18 PepMap TM, 5μm, 5mm * 300μm i.d.).

*Peptide fractionation:* The peptides were loaded and separated on a C18 column (PepMapTM 100, 3 µm, 100Å, 75 µm id x 15 cm) at a flow rate 0.3 μL/min and using a linear binary gradient (solvent A: 0.1% formic acid; solvent B: 80% acetonitrile 0.1% formic acid) as follows: 0-43min, 2-35%B; 43-51min, 35-60%B 51-52min, 60-100%B; 52-61min, 100%B; 62-77min, 2%B.

*MS analyser operational conditions:* The Orbitrap Elite was operated in data-dependent mode, automatically switching between MS and MS2. Full scan MS spectra (300-2,000 m/z) were acquired at 30,000 (m/z 400) resolution using an automatic gain control (AGC) target value of 10e6 charges. The systems were controlled by XCalibur software (version 2.1 SP1.48). Internal mass calibration was performed using (Si(CH_3_)_2_O)_6_H+ m/z 445.120025 as a lock mass. Dynamic exclusion was enabled with exclusion size list of 500 and exclusion duration of 30s. The 10 most intense precursors were selected for subsequent fragmentation. The normalised collision energy was 35%. The fragmentation was acquired in the ion trap with an isolation window of 2.0 m/z, a target value of 5,000, an activation Q of 0.25 and an activation time of 10 ms.

*Spectra analyses:* CID spectra were processed in Mascot using Proteome Discoverer (version 1.4.0.288, Thermo) by searching against the SwissProt Fungi (released on the 21^st^ of January 2013, 2651485 entries) and the NCBI (restricted to fungi, 4487301 entries) databases. The searches were performed with the following parameters: enzyme: trypsin, 2 missed cleavages; mass accuracy precursor: 5 ppm and mass accuracy fragments: 0.5 Da (CID, fixed modifications: carbamidomethyl (C), dynamic modifications: Dioxidation (W), Gln->pyro-Glu (N-term Q), Glu->pyro-Glu (N-term E), Oxidation (HW), Trp-> Kynurenin (W) methionine oxidation. Only proteins following these filters, with peptide confidence> 95%, were added to the list of the identified protein species.
